# How statistically fragile are randomized controlled trials comparing quadriceps tendon autografts with hamstring or bone‐patellar tendon‐bone autografts in anterior cruciate ligament reconstruction?

**DOI:** 10.1002/ksa.12535

**Published:** 2024-11-04

**Authors:** Joshua Dworsky‐Fried, Luca Bernardini, Prushoth Vivekanantha, Lauren Gyemi, Amit Meena, Sachin Tapasvi, Christian Fink, Darren de SA

**Affiliations:** ^1^ Michael DeGroote School of Medicine McMaster University Hamilton Ontario Canada; ^2^ School of Graduate Studies, Faculty of Health Sciences McMaster University Hamilton Ontario Canada; ^3^ Department of Surgery, Division of Orthopaedic Surgery McMaster University Hamilton Ontario Canada; ^4^ Gelenkpunkt—Sports and Joint Surgery FIFA Medical Center of Excellence Innsbruck Austria; ^5^ Research Unit for Orthopedic Sports Medicine and Injury Prevention (OSMI), Private University for Health Sciences Medical Informatics and Technology Innsbruck Austria; ^6^ Department of Orthopedics Shalby Hospital Jaipur India; ^7^ Orthopaedic, Sahyadri Super Specialty Hospital Deccan Gymkhana Pune India

**Keywords:** ACL reconstruction, continuous fragility index, fragility index, reverse fragility index, quadriceps tendon

## Abstract

**Purpose:**

To determine the statistical fragility of randomized controlled trials (RCTs) which compare the use of quadriceps tendon (QT) autografts to either hamstring tendon (HT) or bone‐patellar tendon‐bone (BPTB) autografts in anterior cruciate ligament reconstruction (ACLR).

**Methods:**

A search was conducted across PubMed, MEDLINE and EMBASE databases for RCTs comparing QT autografts to HT or BPTB autografts in ACLR from inception to 21 April 2024. Studies that reported ≥1 statistically significant continuous outcome, statistically significant dichotomous outcome and/or nonsignificant dichotomous outcome were included for analysis. The fragility index (FI), continuous fragility index (CFI) and reverse fragility index (RFI) were calculated for significant dichotomous outcomes, significant continuous outcomes and nonsignificant dichotomous outcomes, respectively.

**Results:**

A total of 11 RCTs comprising 716 patients were included. The mean sample size was 65.8 patients. The median FI among nine outcomes from four studies was 1.0 (interquartile range [IQR], 0.5; 95% confidence interval [CI], 0.6–1.4; range 0.5–1.5). The number of patients lost to follow‐up at the final follow‐up period was more than the study‐specific FI in three (75%) studies. The median CFI among 30 outcomes from six studies was 4.9 (IQR, 10.1, 95% CI, 3.9–8.2; range 0–18.2). The number of patients lost to follow‐up at the final follow‐up period was more than the study‐specific CFI in four (66.7%) studies. The median RFI among 10 outcomes from five studies was 5.0 (IQR, 3.5; 95% CI, 3.4–6.6; range 1.0–9.0). The number of patients lost to follow‐up at the final follow‐up period was more than the study‐specific RFI in four (80%) studies.

**Conclusion:**

This systematic review revealed that regardless of the metric used, RCTs comparing QT autografts to HT or BPTB autograft options in ACLR are statistically fragile. While the indices of statistical fragility evaluated in this study are important metrics of robustness to consider, their application in research and clinical practice needs to be further elucidated.

**Level of Evidence:**

Level I.

**Abbreviations**
ACLR: anterior cruciate ligament reconstruction
BPTB: bone‐patellar tendon‐bone
CFI: continuous fragility index
CI: confidence interval
FI: fragility index
H/Q: strength ratio of hamstrings/quadriceps
HT: hamstring tendon
IQR: interquartile range
LHD: single lateral hop for distance test
LSI: limb ‐symmetry index
MHD: single medial hop for distance test
N/A: not applicable/available
PROM: patient reported outcome measure
QT: quadriceps tendon
QTB: quadriceps tendon with bone
RCT: randomized controlled trial
RFI: reverse fragility index
SD: standard deviation
SHD: single hop for distance test
STG: semitendinosus tendon‐gracilis

## INTRODUCTION

Anterior cruciate ligament (ACL) injuries are one of the most frequently occurring knee injuries in sports, and ACL reconstruction (ACLR) has been well‐established as the gold standard of treatment [15]. Autografts have been demonstrated to be superior to allografts [3] when performing ACLR, with bone‐patellar tendon‐bone (BPTB) and hamstring tendon (HT) autografts being the two most popular options over the past few decades [3, 12, 17]. In recent years, there has been an increased interest in the quadriceps tendon (QT) autograft, which has been reported to have comparable graft survival and functional and stability outcomes to both BPTB and HT autografts [4, 14, 50]. Recent literature has also suggested the superior nature of QT autografts compared to their counterparts with less donor site morbidity and harvest pain than BPTB autografts [14, 22, 50], as well as less knee laxity, lower failure rates and increased outcome scores to HT autografts [9, 41, 42]. Some studies have shown conflicting results regarding QT autografts, therefore it is critical to evaluate a series of studies of higher‐quality evidence [60].

To determine the true validity of QT autografts as an equivalent or superior alternative to BPTB and HT options, the robustness of studies that compare the graft options must be assessed. With randomized controlled trials (RCTs) being the gold standard of evidence‐based medicine [38], one metric that has been developed to evaluate the strength of the trial results is the fragility index (FI) [58]. The FI was first defined by Walsh et al. [58], as the minimum number of patients whose status would have to change from a nonevent to an event required to turn a statistically significant result into a nonsignificant result, with a smaller FI indicating a more fragile result. While the FI can be used to assess dichotomous outcomes [58], the continuous fragility index (CFI) and reverse fragility index (RFI) can be used for continuous outcomes [2] and nonsignificant dichotomous outcomes [27], respectively. A recent review of all RCTs in the *American Journal of Sports Medicine* determined that 29.5% of RCTs that reported statistically significant results had an FI of zero when using a more conservative analysis [52]. Thus, the statistical fragility of orthopaedic trials has been called into question [57]. Despite the recent emergence in popularity of the QT autograft, no reviews to date have assessed the statistical fragility of RCTs comparing the QT autograft with alternative options in ACLR. Assessing the quality of these trials can help guide improvements in methodologies of future trials investigating this topic. Therefore, the aim of this study is to evaluate the robustness of RCTs comparing QT autograft with other graft options in ACLR using metrics including the FI, RFI and CFI. It is hypothesized that the included RCTs will be similar to other trials in sports medicine literature, in that the results will be statistically fragile.

## METHODS

The Preferred Reporting Items for Systematic Reviews and Meta‐Analyses and Revised Assessment of Multiple Systematic Reviews guidelines for coordinating and reporting systematic reviews were followed during the development of this research [30, 33].

### Search criteria

Three online databases (PubMed, MEDLINE, EMBASE) were searched from database inception to 21 April 2024, to identify RCTs comparing QT with HT or BPTB autograft options in ACLR. Comprehensive search terms including ‘quadriceps’, ‘graft’ or ‘autograft’ and ‘anterior cruciate ligament’ or ‘ACL’ were utilized (Supporting Information S1: Digital Material Table 1). Studies were included if they met the following criteria: (1) two‐armed RCTs comparing QT autografts with HT or BPTB autografts, (2) ≥1 statistically significant continuous outcome, statistically significant dichotomous outcome and/or nonsignificant dichotomous outcome, (3) human studies and (4) studies published in the English language. Exclusion criteria were (1) level II‐IV evidence, (2) textbook chapters, (3) conference abstracts and (4) biomechanical or cadaveric/animal studies. References of included studies and pertinent review papers were manually searched to ensure all means of study identification were exhausted.

**Table 1:** Demographic characteristics.

First author (year of publication)	Journal	Detsky Score	Graft comparison	Sample size	Females (%)	Follow‐up time	Lost to follow‐up	Number of outcomes	Number of primary/secondary outcomes	Number of objective/subjective outcomes	Detsky Score
Barié (2020)	*Archives of Orthopaedic and Trauma Surgery*	17/22 (77.27%)	QTB vs. BPTB	60	43	10 years	17	2	0/2	2/0	17/22 (0.7727)
Buescu (2017)	*Acta Orthopaedica et Traumatologica Turcica*	18/21 (85.71%)	QT vs. HT	48	20.8	NR	NR	2	2/0	2/0	18/21 (0.8571)
Ebert (2024)	*The American Journal of Sports Medicine*	19/22 (86.36%)	QT vs. HT	112	50	2 years	15	7	1/6	6/1	19/22 (0.8636)
Horstmann (2022)	*Archives of Orthopaedic and Trauma Surgery*	22/22 (100%)	QT vs. HT	51	35.29	2 years	7 (13.7%)	1	0/1	1/0	22/22 (1)
Lind (2020)	*British Journal of Sports Medicine*	20/22 (90.91%)	QT vs. HT	99	45.45	2 years	8	4	0/4	4/0	20/22 (0.9091)
Lund (2014)	*Arthroscopy: The Journal of Arthroscopic & Related Surgery*	22/22 (100%)	QT vs. HT	51	BPTB: 16% QTB: 19% Total 17.6	2 years	12	7	3/4	7/0	22/22 (1)
Martin‐Alguacil (2019)	*Acta Orthopaedica et Traumatologica Turcica*	18/22 (81.82%)	QT vs. HT	51	23.5	1 year	13	4	0/4	4/0	18/22 (0.8182)
Martin‐Alguacil (2018)	*The Knee*	20/22 (90.91%)	QT vs. HT	56	21.4	2 years	20	6	3/3	6/0	20/22 (0.9091)
Pigozzi (2004)	*Journal of Sports Medicine and Physical Fitness*	13/22 (59.09%)	QT vs. BPTB	48	25	6 months	NR	7	7/0	7/0	13/22 (0.5909)
Sinding (2020)	*Sports Medicine*	20/22 (90.91%)	QT vs. STG	100	37	1 year	15	11	0/11	11/0	20/22 (0.9091)
Tang (2024)	*European Journal of Orthopaedic Surgery & Traumatology*	18/22 (81.82%)	QT vs. HT	40	7.5	1 year	3	2	1/1	2/0	18/22 (0.8182)

Abbreviations: BPTB, bone‐patellar tendon‐bone; HT, hamstring tendon; QT, quadriceps tendon; QTB, quadriceps tendon with bone; STG, semitendinosus tendon‐gracilis.

### Screening

Independent and blinded title and abstract screening were conducted by two authors (J. D. F. and L. B.), with conflicts resolved through consensus or consultation with a more senior author (P. V.). During the full‐text screening stage, studies were independently screened by the initial two authors, and disagreements were resolved in a similar manner.

### Assessment of agreement

The interreviewer agreement was evaluated using Cohen's kappa (*κ*) coefficient statistic for screening. A priori classification was determined using the following criteria: κ of 0.91–0.99 was considered to be almost perfect agreement; κ of 0.71–0.90 was considered to be substantial agreement; κ of 0.61–0.70 was considered to be high agreement; κ of 0.41–0.60 was considered to be moderate agreement; κ of 0.21–0.40 was considered to be fair agreement and a κ value of 0.20 or less was considered to be no agreement [32].

### Quality assessment

The Detsky Quality Assessment Scale was used for the quality assessment of RCTs [16]. The scale contains 14 questions categorized as (1) randomization, (2) outcome measures, (3) inclusion and exclusion criteria and reasons for patient exclusion, (4) interventions and (5) statistical analysis [16]. Equal weight was given to each category and each category could obtain a maximum of four points [16]. If there were negative findings, an additional question was added to the statistical analysis category (i.e., were confidence intervals [CIs] calculated or posthoc power calculations performed?) [16]. Maximum scores for trials with positive and negative findings were 20 and 21 points, respectively [16]. Scores were then made into a percentage.

### Data extraction

Data were extracted in an electronic spreadsheet designed a priori (Google Sheets; Google LLC). Extracted data included study characteristics (i.e., authors, year of publication, level of evidence) and demographic data (i.e., sample size, patient age, sex, etc.), follow‐up time and the number lost to follow‐up. The type of graft used (QT, HT or BPTB) was also recorded. For each significant outcome, sample size and mean outcome values for both groups, as well as *p* values were extracted. Outcomes were categorized as continuous or dichotomous. Additionally, outcomes were categorized as either primary or secondary, as well as either subjective or objective. Subjective outcomes were defined as those interpreted by individual patients, such as patient‐reported outcome measures (PROMs). Objective outcomes were defined as outcomes that are formally assessed including strength, instability rates and return to sport.

### Fragility calculations

#### Calculation of the FI

The FI was calculated using the same method as described by Walsh et al. [28, 58]. A two‐by‐two contingency table was constructed to compare the number of outcome events to the number of outcome nonevents across both groups. Then, to calculate the FI, one event was added to the group with the fewer number of events while simultaneously subtracting a nonevent from the same group to maintain a constant patient population size. A Fisher's exact test was used to determine the two‐tailed *p* value. This process was repeated until the calculated *p* ≥ 0.05, representing a nonsignificant outcome. Therefore, the FI was defined as the number of iterations performed until a nonsignificant *p* value is achieved from Fisher's exact test [58]. The FI was calculated for each significant dichotomous outcome from each included RCT using the method described above.

#### Calculation of CFI

To calculate the CFI, the mean outcome values for significant continuous outcomes across both groups needed to be extracted. Using those values, a Welch *t* test was then conducted. If the resulting *p* value was significant (<0.05), then the data set with the higher mean outcome value was identified. The data point in that data set which was closer to but still greater than the mean is moved to the mean of the lower‐mean data set. Subsequently, another Welch *t* test was conducted. If the resulting *p* value was still significant, this process was repeated until the *p* value obtained from the Welch *t* test became >0.05, representing a nonsignificant difference between the two groups. The CFI was defined as the number of iterations performed until a nonsignificant *p* value was achieved from the Welch *t* test [2, 8].

To calculate the CFI as described above, all data points in the data set of interest were required. However, it is common for RCTs to only report means and standard deviations (SD) of measured outcomes rather than providing the full raw data set. To circumvent this disparity, an iterative substitution algorithm has been described [8] which generates a normally distributed candidate data set for each group to calculate CFI. The CFI values included in this study were all calculated using an online calculator that applies this algorithm and code [8]. To calculate the CFI using this online calculator, values such as sample size, mean and SD across both groups in each study were inputted. The tolerance of the online calculator, which is a measure of how similar the simulated data set must be compared to the RCT outcome values, was set at 0.01. The number of iterations, which is the number of unique data sets simulated to calculate the CFI, was set to 10. The CFI was calculated for each significant continuous outcome from each included RCT using the method described above.

#### Calculation of RFI

The RFI was calculated using a similar method as the FI calculation described above but in the reverse direction. One event was subtracted from the group with a fewer number of events while simultaneously adding a nonevent to the same group to maintain a constant patient population size [27]. A Fisher's exact test was used to determine the two‐tailed *p* value, and this process was repeated until the calculated *p* value became <0.05, representing a significant outcome. Therefore, the RFI is defined as the number of iterations performed until a significant *p* value is achieved from Fisher's exact test [27]. The RFI was calculated for each nonsignificant dichotomous outcome from each included RCT using the method described above.

### Statistics and outcome reporting

Demographic variables were reported as means with associated SDs, 95% CIs or ranges. For each study, FI, CFI and RFIs for each outcome at the latest follow‐up were averaged. These means were then used to calculate an overall median FI, CFI or RFI, with associated interquartile ranges (IQR). Outcomes were considered significant if *p* ≤ 0.05.

## RESULTS

### Study selection and characteristics

**Figure 1:**
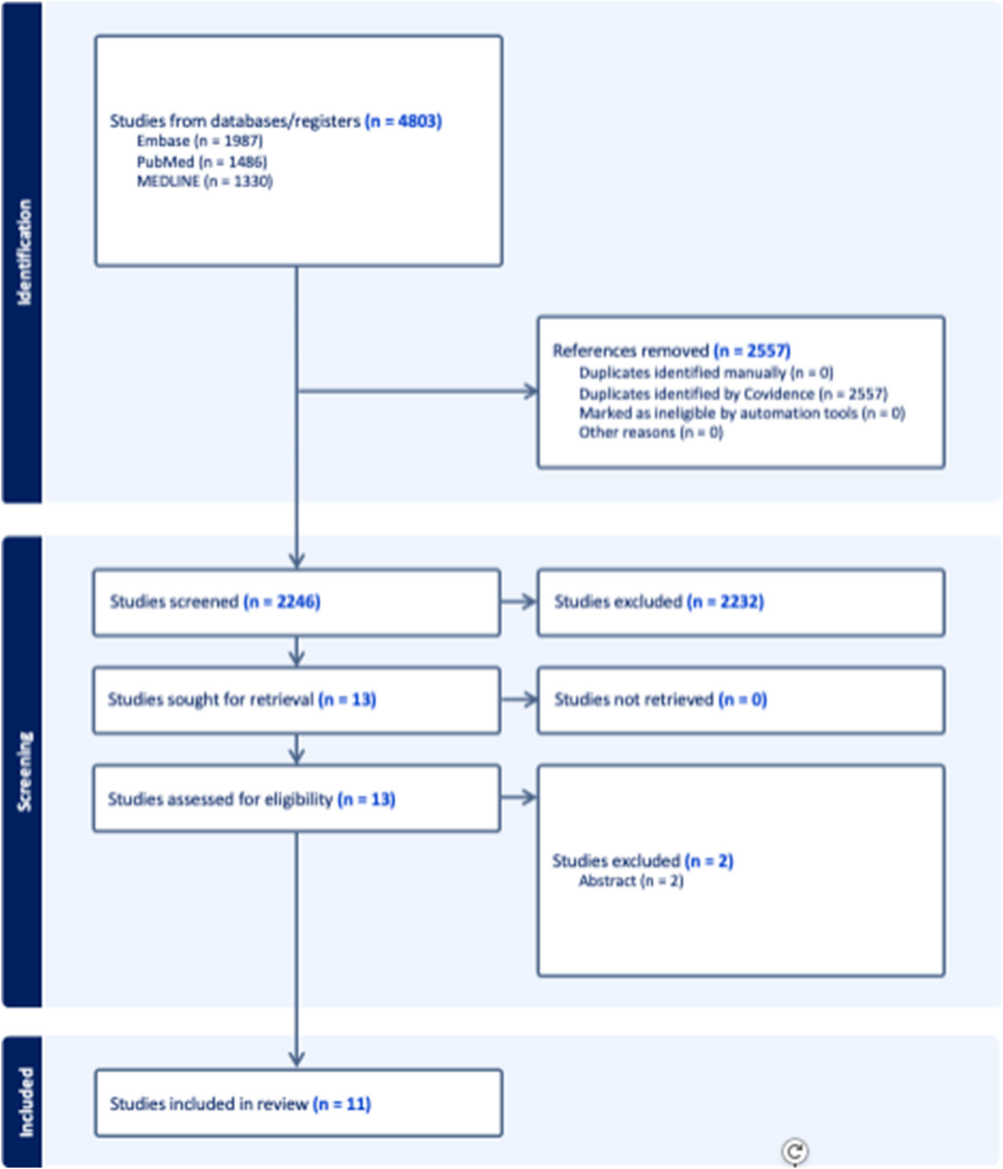
Preferred reporting items for systematic reviews and meta‐analyses flow diagram representing a systematic review comparing quadriceps tendon autografts with hamstring tendon or bone‐patellar tendon‐bone autografts in anterior cruciate ligament reconstruction.

A total of 4803 studies across EMBASE, PubMed and MEDLINE were identified. 2557 duplicate studies were removed, resulting in 2246 studies remaining for title and abstract screening. After applying the aforementioned eligibility criteria, 11 studies were identified and included [5, 7, 18, 23, 34, 35, 39, 40, 47, 53, 55] (Figure 1). The eleven included studies had a total of 716 patients (33.5% female, mean age of 27.6 years [range: 18.7–35.0 years]) before any patients were lost to follow‐up. The mean follow‐up time was 2.4 (SD 2.8) years. Only outcomes that were recorded during the final follow‐up period were included. The mean sample size was 65.8 (SD 25.5). The mean percentage of loss to follow‐up per study, recorded only at the final follow‐up period, was 17.3% (SD 10.7%). A total of 50 individual outcomes were included and analyzed, of which 17 were primary outcomes and 33 were secondary outcomes. Of these 50 individual outcomes, 49 were objective and one was subjective. There was an almost perfect agreement during the title and abstract stage (κ = 0.93, 95% CI, 0.8–1.0) and during the full‐text stage (κ = 1.00). The mean Detsky score for the eleven included studies was 85.9% (SD 11.4%) (Table 1).

### FI

The overall median FI for included RCTs, calculated across nine outcomes from four studies [5, 7, 34, 35] was 1.0 (IQR, 0.5; 95% CI, 0.6–1.4; range, 0.5–1.5) (Table 2). Two primary dichotomous outcomes from a single study [7] were significant, with a median FI of 1.0 (IQR, 1.0, 95% CI, ‐1.0–3.0). Seven secondary dichotomous outcomes from three studies [5, 34, 35] were significant, with a median FI of 2.0 (IQR, 1.0, 95% CI, 0.4–1.9) (Table 2). Nine objective outcomes were identified with a median FI of 1.0 (IQR, 2.0, 95% CI, 0.4–1.8); however, there were no subjective outcomes from the included studies (Table 2). The number of patients lost to follow‐up at the final follow‐up period was more than the study‐specific FI in three (75%) studies [5, 34, 35]. Of these, the number of patients lost to follow‐up was, on average, 11.3 (95% CI, 5.9–16.8) more than the study‐specific FI (Table 2). Five significant dichotomous outcomes from three studies [5, 7, 35] assessed pain and analgesia use, with a median FI of 1.0 (IQR, 2.0; 95% CI, 0.1–2.3). Three function‐related outcomes from two studies [34, 35] had a median FI of 1.0 (IQR, 1.0; 95% CI, −0.1 to 2.1). Only one significant dichotomous outcome assessed instability, with an FI of 1.0 (Table 2) [35]. Overall individual study results can be found in Table 3.

**Table 2:** FI values.

First author (year of publication)	Mean FI overall	Mean FI (primary/secondary outcomes)	Mean FI (objective/subjective outcomes)	Mean CFI (overall)	Mean CFI (primary/secondary outcomes)	Mean CFI (objective/subjective outcomes)	Mean RFI (overall)	Mean RFI (primary/secondary outcomes)	Mean RFI (objective/subjective outcomes)
Barié (2020)	0.5 *p* = 0.019, 0.046	N/A/0.5	0.5/N/A	N/A	N/A/N/A	N/A/N/A	N/A	N/A/N/A	N/A/N/A
Buescu (2017)	1 *p* = 0.009, 0.002	1/N/A	1/N/A	N/A	N/A/N/A	N/A/N/A	N/A	N/A/N/A	N/A/N/A
Ebert (2024)	N/A	N/A/N/A	N/A/N/A	9.98 *p* = 0.014, 0.03, <0.0001, 0.026, 0.046, 0.007	N/A/9.98	11.98/0	6*p* = n.s	6/N/A	6/N/A
Horstmann (2022)	N/A	N/A/N/A	N/A/N/A	N/A	N/A/N/A	N/A/N/A	4 *p* = n.s	N/A/4	4/N/A
Lind (2020)	1 *p* = 0.04	N/A/1	1/N/A	4.75 *p* = 0.02, 0.03, 0.046	N/A/4.75	4.75/N/A	9 *p* = n.s	N/A/9	9/N/A
Lund (2014)	1.5 *p* = 0.03, 0.03, 0.02, <0.05	N/A/1.5	1.5/N/A	N/A/	N/A/N/A	N/A/N/A	6.67 *p* = n.s	6.67/N/A	6.67/N/A
Martin‐Alguacil (2019)	N/A	N/A/N/A	N/A/N/A	N/A/	N/A/N/A	N/A/N/A	2.75 *p* = n.s	N/A/2.75	2.75/N/A
Martin‐Alguacil (2018)	N/A	N/A/N/A	N/A/N/A	0.8 *p* = 0.003, 0.012, 0.005, 0.014, 0.004, 0.04	1.6/0	0.8/N/A	N/A	N/A/N/A	N/A/N/A
Pigozzi (2004)	N/A	N/A/N/A	N/A/N/A	12 *p* < 0.01, 0.05, 0.05, 0.05, 0.05, 0.05, 0.05	12/N/A	16.8/0	N/A	N/A/N/A	N/A/N/A
Sinding (2020)	N/A	N/A/N/A	N/A/N/A	1.8 *p* < 0.01, 0.01, 0.01, 0.01, 0.03, 0.01, 0.01, 0.01, 0.01, 0.01, 0.01	N/A/1.8	1.8/N/A	N/A	N/A/N/A	N/A/N/A
Tang (2024)	N/A	N/A/N/A	N/A/N/A	3.4 *p* < 0.05	3.4/N/A	3.4/N/A	N/A	N/A/N/A	N/A/N/A

Abbreviations: CFI, continuous fragility index; FI, fragility index; N/A, not applicable/available; n.s, not significant; RFI, reverse fragility index.

**Table 3:** Study outcomes.

	Significant dichotomous outcomes (%)			Significant continuous (value [SD])		Nonsignificant dichotomous (%)	
**First author (year of publication)**	**Pain and analgesia**	**Function**	**Instability**	**Strength**	**Function**	**Instability**	**Function**
Barié (2020)	Postoperative pain during kneeling (QTB: 33% BPTB: 64%)	N/A	N/A	N/A	N/A	N/A	N/A
	Postoperative pain during squatting (QTB: 29% BPTB: 55%)	N/A	N/A	N/A	N/A	N/A	N/A
Buescu (2017)	Median number of supplementary analgesic drug administration (QT: 16.7% HT: 37.5%)	N/A	N/A	N/A	N/A	N/A	N/A
	Number who did not receive rescue analgesic (QT: 50% HT: 13%)	N/A	N/A	N/A	N/A	N/A	N/A
Ebert (2024)	N/A	N/A	N/A	Knee extensor torque LSI (QT: 77.3 [15.9] HT: 85.3 [13.1])	ACL‐RSI score (QT: 60.9 [24.7] HT: 68.1 [25.8])	KT‐1000 Knee Arthrometer Side‐to‐Side Difference Scores (QT: 91.7% HT: 93.9%)	N/A
	N/A	N/A	N/A	Knee flexor torque LSI (QT: 101.1 [5.1] HT: 94 [8])	N/A	N/A	N/A
	N/A	N/A	N/A	SHD LSI (QT: 80.4 [16.3] HT: 87.9 [10.2])	N/A	N/A	N/A
	N/A	N/A	N/A	LHD LSI (QT: 86.6 [12.6] HT: 91.9 [9.6])	N/A	N/A	N/A
	N/A	N/A	N/A	MHD LSI (QT: 75.4 [17.6] HT: 85.5 [14.4])	N/A	N/A	N/A
Horstmann (2022)	N/A	N/A	N/A	N/A	N/A	Pivot Shift Grade 0/1 + /2 + /3+ (QT: 23/0/0/0 HT: 26/0/0/0)	N/A
Lind (2020)	N/A	Donor site morbidity (QT: 27% HT: 50%)	N/A	One‐leg hop test % of normal side (QT: 91 [13] HT: 97 [11])	Donor site morbidity score (QT: 14 (17) HT: 22 (18))	Negative pivot shift (QT: 83% HT: 77%)	N/A
Lund (2014)	Harvest site pain (QT: 0% BPTB: 30%)	Positive pivot shift (QT: 14% BPTB: 38%)	Crural sensitivity loss (QT: 48% BPTB: 73%	N/A	N/A	2 mm or less anteroposterior laxity (QT: 77% BPTB: 76%)	N/A
	N/A	Knee walking ability test (QT: 5% BPTB: 34%)	N/A	N/A	N/A	Anteroposterior laxity 3–4 mm (QT: 19% BPTB: 16%)	N/A
	N/A	N/A	N/A	N/A	N/A	Anteroposterior laxity 5 mm+ (QT: 4% BPTB: 8%)	N/A
Martin‐Alguacil (2019)	N/A	N/A	N/A	N/A	N/A	N/A	Practice of crutch from the 4th day (QT: 73.1% HT: 92.0%)
	N/A	N/A	N/A	N/A	N/A	N/A	Practice bicycle from the 3rd week (QT: 57.7% HT: 76%)
	N/A	N/A	N/A	N/A	N/A	N/A	Practice running from the 3rd month (QT: 96.2% HT: 80.0%)
	N/A	N/A	N/A	N/A	N/A	N/A	Practice normal training after 6 months (QT: 34.6% HT: 44.0%)
Martin‐Alguacil (2018)	N/A	N/A	N/A	H/Q ratio 60 deg/s (QT: 120.3 [39.9] HT: 97.6 [16.1])	N/A	N/A	N/A
	N/A	N/A	N/A	H/Q ratio 180 deg/s (QT: 122.5 [38.3] HT: 99.7 [14.4])	N/A	N/A	N/A
	N/A	N/A	N/A	H/Q ratio 300 deg/s (QT: 120.9 [28.8] HT: 103.7 [10.7])	N/A	N/A	N/A
Pigozzi (2004)	N/A	N/A	N/A	Jump test % (QT: 11.4 [1.8] BPTB: 24 [3.2])	N/A	N/A	N/A
	N/A	N/A	N/A	Leg press 3 repetitions peak torque (QT: 8.4 [2.1] BPTB: 15.2 [3.4])	N/A	N/A	N/A
	N/A	N/A	N/A	Leg press 3 repetitions total work (QT: 8.9 [2.4] BPTB: 14.4 [4.1])	N/A	N/A	N/A
	N/A	N/A	N/A	Knee extension 3 repetitions peak torque (QT: 17.6 [3.5] BPTB: 30.3 [5.1])	N/A	N/A	N/A
	N/A	N/A	N/A	Knee extension 3 repetitions total work (QT: 16.5 [2.9] BPTB: 26.4 [4.5])	N/A	N/A	N/A
	N/A	N/A	N/A	Knee flexion 3 repetitions peak torque (QT: 8.6 [2.4] BPTB: 14.1 [3.1])	N/A	N/A	N/A
	N/A	N/A	N/A	Knee flexion 3 repetitions total work (QT: 9.4 [2.8] BPTB: 11.6 [2.1])	N/A	N/A	N/A
Sinding (2020)	N/A	N/A	N/A	Knee extension ‐60 deg/s (QT: 2.77 [0.74] STG: 2.95 [0.66])	N/A	N/A	N/A
	N/A	N/A	N/A	Knee extension 0 deg/s (QT: 2.39 [0.76] STG: 2.69 [0.64])	N/A	N/A	N/A
	N/A	N/A	N/A	Knee extension 60 deg/s (QT: 2.13 [0.63] STG: 2.33 [0.49])	N/A	N/A	N/A
	N/A	N/A	N/A	Knee extension 180 deg/s (QT: 1.56 [0.48] STG: 1.66 [0.40])	N/A	N/A	N/A
	N/A	N/A	N/A	Knee extension RFD 50 ms (QT: 22.2 [8.7] STG: 23.0 [11.7])	N/A	N/A	N/A
	N/A	N/A	N/A	Knee extension RFD 200 ms (QT: 9.8 (4.0) STG: 10.2 (2.7))	N/A	N/A	N/A
	N/A	N/A	N/A	Knee flexion 0 deg/s (QT: 1.76 [0.48] STG: 1.65 [0.40])	N/A	N/A	N/A
	N/A	N/A	N/A	Knee flexion 60 deg/s (QT: 1.56 [0.42] STG: 1.36 [0.36])	N/A	N/A	N/A
	N/A	N/A	N/A	Knee flexion 180 deg/s (QT: 0.94 (0.28) STG: 0.78 (0.22))	N/A	N/A	N/A
	N/A	N/A	N/A	Knee flexion RFD 50 ms (QT: 12.3 [6.1] STG: 9.9 [3.7])	N/A	N/A	N/A
	N/A	N/A	N/A	Knee flexion RFD 200 ms (QT: 6.8 [2.8] STG: 5.4 [1.6])	N/A	N/A	N/A
Tang (2024)	N/A	N/A	N/A	H/Q ratio (QT: 17 HT: 16)	N/A	N/A	N/A

Abbreviations: ACL‐RSI, anterior cruciate ligament return to sports after injury scale; BPTB, bone‐patellar tendon‐bone; H/Q, strength ratio of hamstrings/quadriceps; HT, hamstring tendon; LHD, single lateral hop for distance test; LSI, Limb Symmetry Index; MHD, single medial hop for distance test; N/A, not applicable/available; QT, quadricep tendon; QTB, quadricep tendon with bone; SD, standard deviation; SHD, single hop for distance test; STG, semitendinosus tendon‐gracilis.

### CFI

The overall median CFI for included RCTs among 30 outcomes from six studies [18, 34, 40, 47, 53, 55] was 4.9 (IQR, 10.1, 95% CI, 3.9–8.2; range 0–18.2) (Table 2). Eleven primary continuous outcomes from three studies [40, 47, 55] were significant, with a median CFI of 9.1 (IQR, 9.3; 95% CI, 5.0–11.7). Nineteen secondary continuous outcomes from three studies [18, 34, 53] were significant, with a median CFI of 3.0 (IQR, 6.8, 95% CI, 2.2–7.2). Twenty‐nine objective continuous outcomes from six studies [18, 34, 40, 47, 53, 55] were significant, with a median CFI of 5.8 (IQR, 10.2, 95% CI, 4.1–8.4). One subjective continuous outcome from one study [18] was significant, with a CFI of 0. The number of patients lost to follow‐up at the final follow‐up period was more than the study‐specific CFI in four (66.7%) studies [18, 34, 40, 53]. Of these, the number of patients lost to follow‐up was, on average, 10.2 (95% CI, 2.9–17.4) more than the study‐specific CFI. The median CFI was 6.0 (IQR, 10.5; 95% CI, 4.2–8.6) for 28 outcomes assessing various measures of strength from five studies [18, 40, 47, 53, 55] and 1.5 (IQR, 1.5; 95% CI, −1.4 to 4.4) among two function‐related outcomes from one study [34]. The overall individual study results can be found in Table 3.

### RFI

The overall median RFI for included RCTs among 10 outcomes from five studies [18, 23, 34, 35, 39] was 5.0 (IQR, 3.5; 95% CI, 3.4–6.6; range 1.0–9.0) (Table 2). Four primary nonsignificant dichotomous outcomes from two studies [18, 35] were identified, with a median RFI of 6.5 (IQR, 1.5; 95% CI, 5.2–7.8). Six secondary nonsignificant dichotomous outcomes from three studies [23, 34, 39] were identified, with a median RFI of 3.5 (IQR, 2.0, 95% CI, 1.7–6.3). All 10 of the nonsignificant dichotomous outcomes were categorized as objective outcomes. The number of patients lost to follow‐up at the final follow‐up period was more than the study‐specific RFI in four (80%) studies [18, 23, 35, 39]. Of these, the number of patients lost to follow‐up was, on average, 7.0 (95% CI, 3.6–10.3) more than the study‐specific RFI. Six nonsignificant dichotomous outcomes from four studies [18, 23, 34, 35] assessed measures of instability, with a median RFI of 6.5 (IQR, 2.5; 95% CI, 5.0–8.0). The median RFI for four function‐related outcomes from one study [39] was 2.5 (IQR, 1.8; 95% CI, 1.1–4.4). Overall individual study results can be found in Table 3.

## DISCUSSION

The primary finding of this study was that the median FI, CFI and RFI for RCTs comparing QT autografts to HT and BPTB autografts in ACLR were 1.0, 4.9 and 5.0, respectively. These values represent the number of patients that would be needed to reverse the statistical significance of the outcomes included for each metric assessed. There is no established consensus on what constitutes a normal or average FI value; however, a common interpretation is that the study may be considered to be fragile if the FI value is equal to or less than the number of patients lost to follow‐up. For the outcomes that were assessed to calculate FI, CFI and RFI, a greater number of patients were lost to follow‐up than would be needed to reverse the significance of the given outcome in 75%, 66.7% and 80% of RCTs included in this study, respectively. Furthermore, regardless of the metric used, the overall median statistical fragility values of studies comparing QT autograft to HT or BPTB autograft in ACLR was five or less, indicating a relatively higher statistical fragility. In addition, the current RCTs comparing QT autografts with other autografts have not found many statistically significant differences in primary or subjective outcomes as compared to secondary or objective outcomes.

RCTs included in this review were, on average, comparable in robustness when measured by statistical fragility, with other trials in sports medicine literature. Previous studies have reported low median FI values (ranging from 2 to 4) [13, 19, 26, 29, 45] and higher median CFI values (ranging from 5 to 14) [1, 2, 8, 21, 44, 59]. The median RFI value of five calculated in this study indicates a higher measure of robustness as compared to the median RFI of three reported in a systematic review comparing HT and BTB autografts in ACLR [48]. These figures suggest that the statistical fragility of the literature evaluating QT autografts in ACLR is comparable to established findings in similar studies. It is important to consider that while these summary metrics give an overall assessment of the statistical fragility, there was a wide range of fragility values and CIs among the RCTs included in our analysis suggesting a variation in their robustness for varying outcomes.

While these are important metrics to consider, caution in their interpretation is warranted as they may not accurately reflect the overall fragility of a given study. Many orthopaedic surgery and sports medicine systematic reviews often suggest that if data from patients lost to follow‐up were to be included in the analysis, the statistical significance of the outcomes would be reversed [2, 13, 29, 46, 51]. However, this is not always the case. As described by Oeding et al. [43], the direct comparison between indices of statistical fragility and the number of patients lost to follow‐up is inherently flawed. The comparison is statistically incompatible, resulting in inaccurate claims misinterpreting the clinical significance of FI, CFI and RFI values [43]. In adding the number of patients lost to follow‐up into the analysis, it must be considered that the patients would have an equal chance of being from the control group as the treatment group; [43] when taking this into account, adding the patients that were lost to follow‐up does not necessarily reverse the significance of the outcomes [43]. Additionally, there are many factors that influence the statistical significance of a study's outcome, (i.e., sample size, number of patients excluded and lost to follow‐up, bias, etc.). Furthermore, there is a lack of consensus on the practical application of these statistical research methods [43]. No single measure of fragility should be used to wholly determine a study's robustness; instead, they should help contribute to a holistic assessment of the robustness of study conclusions [43].

As FI becomes an increasingly relevant measure of robustness, it is important to recognize ways to improve FI values. An umbrella review of FI scores in surgical RCTs determined that the variables associated with increasing FI values are decreasing *p* values and increasing sample sizes and event numbers [57]. The expectation is that larger sample sizes will generate more confidence in the *p* value; [57] however, it is recognized that recruiting patients for surgical trials is known to be difficult [10, 37]. Consequently, it is important to understand measures to increase the number of included patients such as multicentre collaborations, scrutiny of a priori sample size calculations and innovative trial design [57]. Though complex to carry out [11], multicentre trials can be an extremely effective strategy if the method of randomization is prioritized [54]. Additionally, cluster RCTs can be an innovative trial design which has been effective in surgical orthopaedic studies [20, 57]. Loss to follow‐up has also been a factor in decreased sample sizes in surgical RCTs. One article has identified preventative measures to minimize loss to follow‐up, such as ensuring full comprehension of trial expectations, providing monetary incentives and only administering payments after completion of follow‐ups [36]. Alternative strategies may include more flexibility in the follow‐up schedule, routine verification of patients' contact information and providing information to the patients regarding loss to follow‐up and its importance [36]. Furthermore, demographically, females are more likely to visit their doctors than men [6], whereas only 33.5% of the patients in the eleven studies included in this systematic review were female. Thus, the fragility of these studies may have been improved with a more even gender distribution. The importance of reporting FI alongside values of significance has become increasingly emphasized in orthopaedic research to demonstrate the robustness of the findings [31].

Among the 11 studies included in this review, many of them failed to find a significant difference in the primary outcomes they were assessing. Among the significant outcome groups (FI and CFI), only 33.3% of the outcomes were primary. The majority of primary outcomes included in these 11 studies were nonsignificant continuous, thus they were not included in the FI, CFI or RFI calculations. This draws interest as studies tend to be powered toward achieving a significant difference between primary outcomes as opposed to secondary ones [56]. Among the primary outcomes, most studies assessed outcomes such as pain and strength (Table 2), but did not assess those which were significant in comparable level II and III studies such as graft failure rates [24, 49]. In order to promote further robustness among RCTs comparing QT autografts to alternative options, studies should aim to investigate a comprehensive set of outcomes, including PROMs, return‐to‐sports rates, rates of instability, graft failure and strength.

Strengths of the present study include an extensive systematic search of three medical databases and the use of independent, blinded authors to screen and extract data. Furthermore, an almost perfect agreement between the authors was noted during the title, abstract and full‐text stages of screening. RCTs included in this review were of high quality (level I), with a comparable mean Detsky score (85.9%) to that reported in a systematic review of RCTs published in *The Journal of Bone and Joint Surgery* (85%) [25]. Moreover, many orthopaedic‐focused studies only focus on FI which is typically limited to only including significant dichotomous outcomes. The present study included three different metric calculations of statistical fragility by analyzing significant dichotomous, significant continuous and nonsignificant dichotomous outcomes.

This study has several limitations. The inherent practical applicability of FI, CFI and RFI is currently lacking owing to the absence of consensus or established guidelines used to interpret these measures of statistical fragility. Though these measures do provide valuable information, they should not be used independently to determine the robustness of a given study. Additionally, due to the large variability in follow‐up intervals (range 0–10 years), the present study only analyzed clinical outcomes during the final follow‐up period, which may have influenced our findings.

## CONCLUSION

This systematic review showed that regardless of the metric used, RCTs comparing QT autografts to other autograft options in ACLR are statistically fragile. Among these studies, the median FI, CFI and RFI were relatively low when compared to the number of patients lost to follow‐up, indicating high relative fragility. While these are important statistical metrics of robustness, their application in research and clinical practice needs to be further elucidated.

## AUTHOR CONTRIBUTIONS

**Joshua Dworsky Fried**: Screening; data extraction; writing; editing. **Luca Bernardini**: Screening; data extraction; writing; editing. **Prushoth Vivekanantha**: Writing; editing; idea conception. **Lauren Gyemi**: Writing; editing. **Amit Meena**: Writing; editing. **Sachin Tapasvi**: Writing; editing. **Christian Fink**: Writing; editing. **Darren de SA**: Writing; editing; idea conception.

## CONFLICT OF INTEREST STATEMENT

The authors declare no conflict of interest.

## ETHICS STATEMENT

There are no relevant ethical disclosures pertaining to research involving human participants and/or animals, and informed consent was not necessary to develop this manuscript.

## Supporting information

Supporting information.

## Data Availability

Data may be made available upon reasonable request at prushoth.vivekanantha@medportal.ca.
