# Supplementary material for: How statistically fragile are randomized controlled trials comparing quadriceps tendon autografts with hamstring or bone‐patellar tendon‐bone autografts in anterior cruciate ligament reconstruction?
Source: Knee Surg Sports Traumatol Arthrosc. 2024 Nov 4;33(8):2729–44. doi: 10.1002/ksa.12535 (PMC12310093; doi:10.1002/ksa.12535)
Supplement: Supplementary file 1 — Supporting information. [file KSA-33-2729-s001.docx]

**SUPPLEMENTARY DIGITAL MATERIAL:**

**Supplementary Table 1.** Search Criteria

| **EMBASE (n=1987)** | **PUBMED (n=1486)** | **MEDLINE (n=1330)** |
| --- | --- | --- |
| 1. Quadricep | 1. Quadricep | 1. Quadricep |
| 1. quad | 1. quad | 1. quad |
| 1. Graft | 1. Graft | 1. Graft |
| 1. autograft | 1. autograft | 1. autograft |
| 1. Anterior cruciate ligament | 1. Anterior cruciate ligament | 1. Anterior cruciate ligament |
| 1. ACL | 1. ACL | 1. ACL |
| 1. (1 OR 2) AND (3 OR 4) AND (5 OR 6) | 1. (1 OR 2) AND (3 OR 4) AND (5 OR 6) | 1. 1 OR 2) AND (3 OR 4) AND (5 OR 6) |
